# Improvement of Cardiac Function and Subcellular Defects Due to Chronic Diabetes upon Treatment with Sarpogrelate

**DOI:** 10.3390/jcdd11070215

**Published:** 2024-07-09

**Authors:** Paramjit S. Tappia, Vijayan Elimban, Anureet K. Shah, Ramesh K. Goyal, Naranjan S. Dhalla

**Affiliations:** 1 Institute of Cardiovascular Sciences, and Asper Clinical Research Institute, St. Boniface Hospital, Winnipeg, MB R2H 2A6, Canada; ptappia@sbrc.ca; 2 Institute of Cardiovascular Sciences, St. Boniface Hospital Albrechtsen Research Centre, Winnipeg, MB R2H 2A6, Canada; velimban@sbrc.ca; 3 Department of Physiology and Pathophysiology, Max Rady College of Medicine, University of Manitoba, Winnipeg, MB R3E 0J9, Canada; 4 Department of Nutrition and Food Sciences, California State University, Los Angeles, CA 90032, USA; akaur23@calstatela.edu; 5 Department of Pharmacology, Delhi Pharmaceutical Sciences and Research University, New Delhi 110017, India; goyalrk@gmail.com

**Keywords:** diabetic cardiomyopathy, sarpogrelate therapy, subcellular defects, metabolic abnormalities, oxidative stress

## Abstract

In order to investigate the subcellular mechanisms underlying the beneficial effects of sarpogrelate—a 5-HT_2A_ receptor antagonist—on diabetic cardiomyopathy, diabetes was induced in rats by injecting streptozotocin (65 mg/kg). Diabetic animals were treated with or without sarpogrelate (5 mg/kg daily) for 6 weeks; diabetic animals were also treated with insulin (10 units/kg daily) for comparison. Elevated plasma levels of glucose and lipids, depressed insulin levels, hemodynamic alterations and cardiac dysfunction in diabetic animals were partially or fully attenuated by sarpogrelate or insulin treatment. Diabetes-induced changes in myocardial high-energy phosphate stores, as well as depressed mitochondrial oxidative phosphorylation and Ca^2+^-uptake activities, were significantly prevented by these treatments. Reductions in sarcolemma Na^+^-K^+^ ATPase, Na^+^-Ca^2+^ exchange, Ca^2+^-channel density and Ca^2+^-uptake activities were also attenuated by treatments with sarpogrelate and insulin. In addition, decreases in diabetes-induced sarcoplasmic reticulum Ca^2+^-uptake, Ca^2+^-release and Ca^2+^-stimulated ATPase activities, myofibrillar Mg^2+^-ATPase and Ca^2+^-stimulated ATPase activities, and myosin Mg^2+^-ATPase and Ca^2+^-ATPase activities were fully or partially prevented by sarpogrelate and insulin treatments. Marked alterations in different biomarkers of oxidative stress, such as malondialdehyde, superoxide dismutase and glutathione peroxidase, in diabetic hearts were also attenuated by treating the animals with sarpogrelate or insulin. These observations suggest that therapy with sarpogrelate, like that with insulin, may improve cardiac function by preventing subcellular and metabolic defects as a consequence of a reduction in oxidative stress.

## 1. Introduction

It is now well known that chronic diabetes is associated with diabetic cardiomyopathy, which is generally characterized by the development of cardiac hypertrophy, metabolic defects, ultrastructural abnormalities and myocardial fibrosis [1,2,3,4,5,6,7,8]. Although prolonged hyperglycemia and hyperlipidemia due to insulin deficiency or insulin resistance are the major causes of diabetic cardiomyopathy, mitochondrial (MT) dysfunction and sarcoplasmic reticulum (SR) stress have also been indicated to play crucial roles in the pathogenesis of this devastating health hazard [9,10,11,12]. In particular, oxidative stress, inflammation, Ca^2+^-handling abnormalities and apoptosis are pivotal processes that determine the occurrence of cardiac remodeling and cellular injury in diabetic cardiomyopathy [13,14,15,16,17]. In fact, several interventions, such as antihyperglycemic, antioxidant, anti-inflammatory, antifibrotic and antiapoptotic agents, have been shown to exert beneficial effects in preventing diabetic cardiomyopathy [5,7,8,11,12,13,14,15,16,17]. It should also be mentioned that diabetes is not only associated with insulin deficiency or resistance but also found to affect several endocrine, neuronal and other systems, which result in the elevation of hormones, including norepinephrine, angiotensin II, endothelin, serotonin (5-HT) and thyroid hormones [18,19]. Such hormonal imbalances have been reported to promote the development of cardiac remodeling, subcellular alterations, cardiac dysfunction and heart failure in chronic diabetes [18,19]. 

In view of the elevated levels of circulating 5-HT due to the enhancement of platelet aggregation in diabetes [20,21,22,23], sarpogrelate, a 5-HT_2A_ receptor antagonist, has been shown to exert beneficial effects in attenuating a wide variety of abnormalities in chronic diabetes [24,25,26,27,28,29,30,31]. Previously, we observed that the treatment of diabetic animals with sarpogrelate improved cardiac function by promoting the expression of membrane glucose transporters and releasing insulin from the pancreas [32]. Since cardiac dysfunction in chronic diabetes is considered to be intimately associated with the remodeling of subcellular organelles such as the sarcolemma (SL), SR, MT and myofibrils (MFs) [18,19], the present study was undertaken to examine the effects of sarpogrelate on diabetes-induced changes in subcellular activities. In addition, the hemodynamic parameters and metabolic status of diabetic animals with and without sarpogrelate treatment were evaluated by monitoring the plasma levels of glucose and lipids, as well as myocardial high-energy phosphate content and cardiac function. Furthermore, the mechanism of action of sarpogrelate was investigated by measuring the levels of some biomarkers of oxidative stress in diabetic animals with and without sarpogrelate treatment. Since the beneficial actions of sarpogrelate treatment in diabetic cardiomyopathy were similar to those of insulin treatment [32], the effects of insulin treatment on subcellular remodeling in diabetic animals were also studied for the purpose of comparison with sarpogrelate.

## 2. Materials and Methods

### 2.1. Animal Model and Hemodynamic Assessment

Male Sprague-Dawley rats weighing 225–250 g were made diabetic by injecting streptozotocin (65 mg/kg) into the tail vein according to the method described by our laboratory [33]. One week after inducing diabetes, diabetic animals were treated with sarpogrelate (5 mg/kg daily, orally by gavage) or insulin (10 units/kg daily, subcutaneously) for a period of 6 weeks. Human insulin was from Eli Lilly Canada, Toronto, Ontario. The doses and duration of the sarpogrelate and insulin treatments of diabetic animals are similar to those used previously [32]. Sham-treated rats served as a control. All animals were maintained on normal chow and water ad libitum. In one group of animals, blood was drawn from the tail vein; plasma was separated immediately and stored at −20 °C for biochemical analysis. Plasma concentrations of glucose and insulin were determined by using the Worthington Statzyme Reagent Kit (Lakewood, NJ, USA) and radioimmunoassay techniques (Amersham), respectively. For the measurements of plasma triglycerides and cholesterol, enzymatic calorimetric kits (Sigma triglyceride Reagent Kit and Sigma Cholesterol Reagent kit) from Sigma Chemical, St. Louis, MO, USA, were used. Nonesterified free fatty acid levels were determined by using a calorimeter kit (Wako, Osaka, Japan). In another group, the animals were anesthetized with ketamine (90 mg/kg) and xylazine (10 mg/kg), and cardiac performance was measured by inserting a microtip pressure transducer (model 5PR-249, Miller instruments, Houston, TX, USA) into the left ventricle (LV). Heart rate, blood pressure, LV developed pressure (LVDP), LV end-diastolic pressure (LVEDP), the rate of contraction (+dP/dt), and the rate of relaxation (−dP/dt) were recorded. The methods for the hemodynamic assessments of animals as well as for blood analysis are the same as those employed in our laboratory [32]. 

### 2.2. Biochemical and Subcellular Studies

After the termination of hemodynamic studies, the chests of the anesthetized animals were opened under artificial respiration, the hearts were frozen by a Wollenberger clamp precooled in liquid N_2_, and the high-energy phosphate compounds (creatine phosphate, ATP, ADP and AMP) were determined, as described earlier [34]. In all other experiments, rats were decapitated, and the left ventricles, including the septa, were frozen in liquid N_2_ for subcellular and biochemical studies. SL membranes were isolated from the control and experimental hearts according to the procedure established in our laboratory [35]. Methods for the determination of SL Na^+^-K^+^ ATPase, Mg^2+^-ATPase, Ca^2+^-stimulated ATPase, ATP-dependent Ca^2+^-uptake and Na^+^-Ca^2+^ exchange activities were the same as used previously [35]. The SL Ca^2+^-channel density and affinity (1/Kd) were determined by the Scatchard plot analysis of ^3^H-nitrendipine binding data with crude membranes [36]. The methods for the isolation of the cardiac SR and measurement of Ca^2+^-stimulated ATPase, Mg^2+^-ATPase, Ca^2+^-uptake and Ca^2+^-release activities were similar to those described elsewhere [37]. Both MT and MFs were prepared from the cardiac tissue according to procedures used earlier [38,39]. Different MT respiratory and oxidative phosphorylation parameters, as well as Ca^2+^-uptake and Mg^2+^ -ATPase activities, were determined by methods described previously [38]. Cardiac MF Ca^2+^-stimulated ATPase and MF Mg^2+^-ATPase activities were measured [39]. Myosin Mg^2+^-ATPase and Ca^2+^-ATPase activities were also determined according to procedures indicated elsewhere [40,41]. Some biomarkers of oxidative stress, such as malondialdehyde, reduced glutathione, oxidized glutathione, glutathione peroxidase, superoxide dismutase and catalase, were also monitored in the hearts of both the control and experimental groups [42,43,44]. The data are expressed as mean ± SE and compared (control versus diabetic group, diabetic versus sarpogrelate-treated diabetic group, and diabetic versus insulin-treated group) statistically by using the unpaired Student “*t*” test. Statistical differences between multiple groups were evaluated using analysis of variance (ANOVA) followed by Duncan’s new multiple test. A *p* value < 0.05 was considered significant. It should be mentioned that our preliminary experiments showed no significant effects of sarpogrelate or insulin treatment on hemodynamic parameters in control animals.

## 3. Results

### 3.1. Metabolic and Hemodynamic Characteristics

In one series of experiments, the metabolic and hemodynamic profiles of control animals as well as diabetic animals with and without insulin or sarpogrelate treatment were examined, and the data are shown in Table 1 and Table 2, respectively. Untreated diabetic animals showed marked reductions in body weight, heart weight and plasma insulin levels. On the other hand, the plasma levels of glucose, cholesterol, free fatty acids and triglycerides were markedly elevated in diabetic animals (Table 1). All of these alterations in diabetic animals were fully or partially (but significantly) attenuated upon treatment with insulin or sarpogrelate (Table 1). Although the beneficial effects of insulin treatment were somewhat greater than those observed with sarpogrelate, such differences were not significant (*p* > 0.05).

The data in Table 2 show an increase in blood pressure and decreases in heart rate, LVDP, +dP/dt and −dP/dt values in diabetic animals. These alterations in blood pressure and cardiac performance in diabetic animals were significantly suppressed upon treatment with either insulin or sarpogrelate; however, the beneficial effects of these treatments were not significantly different from each other (*p* > 0.05). Furthermore, no differences in LVED values in untreated or treated diabetic animals were observed in comparison to the control animals (Table 2). 

### 3.2. Cardiac Energy Stores and MT Activities

Significant decreases in both creatine phosphate and ATP contents and significant increases in both ADP and AMP contents were observed in untreated diabetic hearts in comparison to those in the control hearts (Table 3). Alterations in all of these parameters were either fully or partially prevented by treatment with either insulin or sarpogrelate. These effects of insulin or sarpogrelate treatment on diabetic animals were not different (*p* > 0.05) from each other (Table 3). In another experiment, the status of MT oxidative phosphorylation, respiratory and some biochemical activities was examined in the control and experimental groups, and the results are shown in Table 4. It can be seen that no changes in respiratory state 4 or the ADP/O ratio were seen in control or diabetic hearts with and without insulin or sarpogrelate treatment. On the other hand, significant decreases in respiratory state 3 and the oxidative phosphorylation rate were observed in diabetic hearts. Likewise, MT Ca^2+^-uptake and Mg^2+^-ATPase activities were depressed in the diabetic heart (Table 4). The treatment of diabetic animals with insulin or sarpogrelate was found to fully or partially (but significantly) attenuate the observed changes in MT state 3 respiration, the oxidative phosphorylation rate and Ca^2+^-uptake activities; however, there was no significant (*p* > 0.05) difference between the beneficial effects of these treatments. It is noted that the depression of MT Mg^2+^-ATPase activity in diabetic hearts was not affected by insulin or sarpogrelate treatment (Table 4). Although the exact reason for the ineffectiveness of these treatments on MT Mg^2+^-ATPase activity is not clear, the possibility of an irreversible change in Mg^2+^-ATPase due to chronic diabetes cannot be ruled out.

### 3.3. Cardiac SL ATPase, Ca^2+^-Uptake and Ca^2+^-Channel Activities

**Figure 1:**
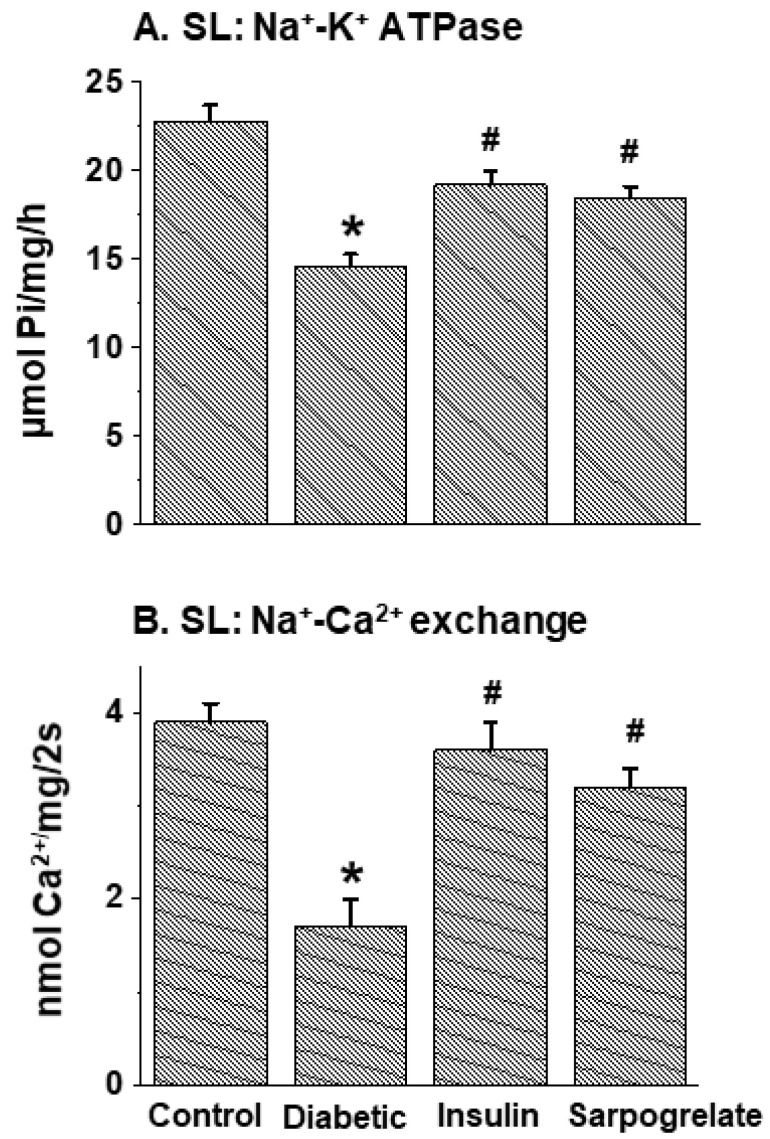
Effects of insulin and sarpogrelate treatments on cardiac sarcolemma (SL) Na^+^-K^+^ ATPase and Na^+^-Ca^2+^ exchange activities in diabetic animals. One week after inducing diabetes with streptozotocin (65 mg/kg), rats were treated with insulin (10 units/kg daily) or sarpogrelate (5 mg/kg daily) for 6 weeks. Values are mean ± SE of 4 experiments in each group. *—*p* < 0.05 vs. control; ^#^—*p* < 0.05 vs. diabetic.

Cardiac SL Na^+^-K^+^ ATPase, Na^+^-Ca^2+^ exchange, ATP-dependent Ca^2+^-uptake, Ca^2+^-stimulated ATPase, Mg^2+^-ATPase and Ca^2+^-channel activities in the control and diabetic animals with and without insulin or sarpogrelate treatment were examined, and the data are given in Figure 1 and Table 5. The activities of Na^+^-K^+^ ATPase, Na^+^-Ca^2+^ exchange, Ca^2+^ uptake and Ca^2+^-stimulated ATPase were depressed in the diabetic heart and were fully or partially (but significantly) attenuated by insulin or sarpogrelate treatment (Figure 1 and Table 5A). The SL Mg^2+^-ATPase activities in diabetic hearts with and without insulin or sarpogrelate treatment were not significantly (*p* > 0.05) different from the control values (Table 5A). Both the SL Ca^2+^-channel density and kd value (dissociation constant) were also decreased in the diabetic heart; these alterations were fully or partially (but significantly) attenuated by the treatment of diabetic animals with insulin or sarpogrelate (Table 5A). It is pointed out that since Ca^2+^-channel affinity is represented by the 1/kd value, it is evident that Ca^2+^-channel affinity is increased in the diabetic heart.

### 3.4. Cardiac SR ATPase, Ca^2+^-Uptake and Ca^2+^-Release Activities

**Figure 2:**
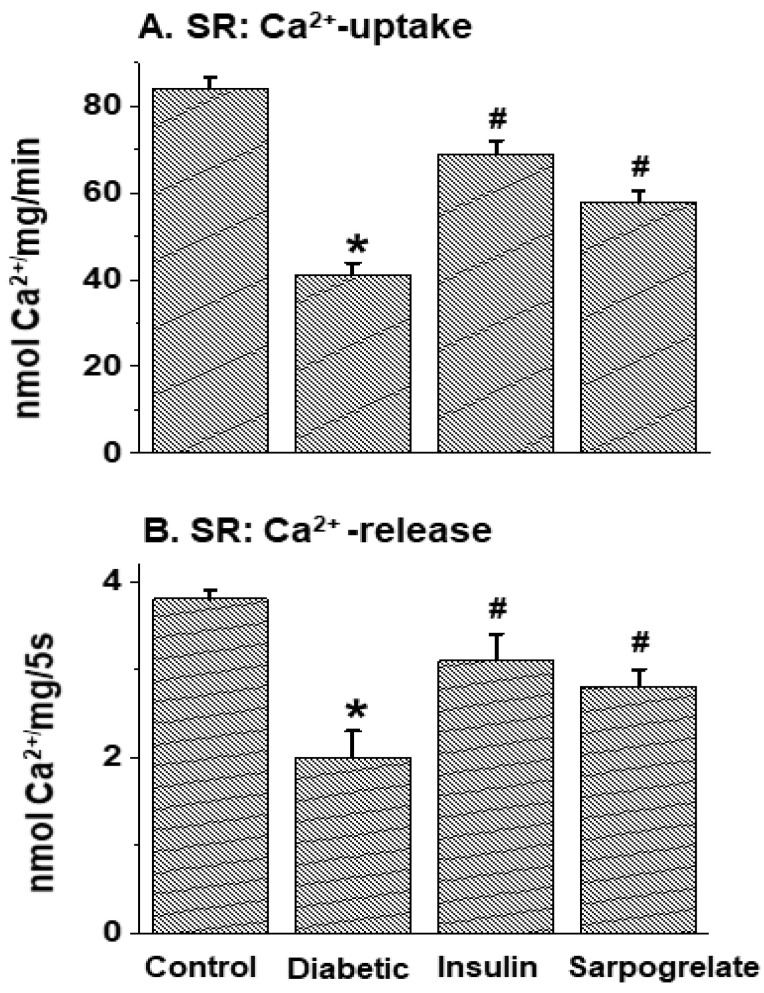
Effects of insulin and sarpogrelate treatments on cardiac sarcoplasmic reticulum (SR) Ca^2+^-uptake and Ca^2+^-release activities in diabetic animals. One week after inducing diabetes, rats were treated with insulin (10 units/kg daily) or sarpogrelate (5 mg/kg daily) for 6 weeks. Values are mean ± SE of 4 experiments in each group. *—*p* < 0.05 vs. control; ^#^—*p* < 0.05 vs. diabetic.

The activities of cardiac SR vesicles obtained from the control and diabetic animals with and without insulin or sarpogrelate treatment were determined, and the data are shown in Table 5B and Figure 2. SR Ca^2+^-stimulated ATPase, Ca^2+^-uptake and Ca^2+^-release activities, unlike Mg^2+^-ATPase activity, were decreased in the diabetic heart (Table 5B and Figure 2). The depression of Ca^2+^-stimulated ATPase activity was fully prevented (Table 5B), whereas the depression of both Ca^2+^-uptake and Ca^2+^-release activities was partially (but significantly) attenuated (Figure 2), but Mg^2+^-ATPase activity (Table 5B) was not affected by insulin or sarpogrelate treatment. Although the beneficial effect of insulin treatment on SR Ca^2+^-release activity was somewhat greater than that of sarpogrelate treatment, the differences were not significant (*p* > 0.05) (Figure 2).

### 3.5. Cardiac MF and Myosin ATPase Activities

**Figure 3:**
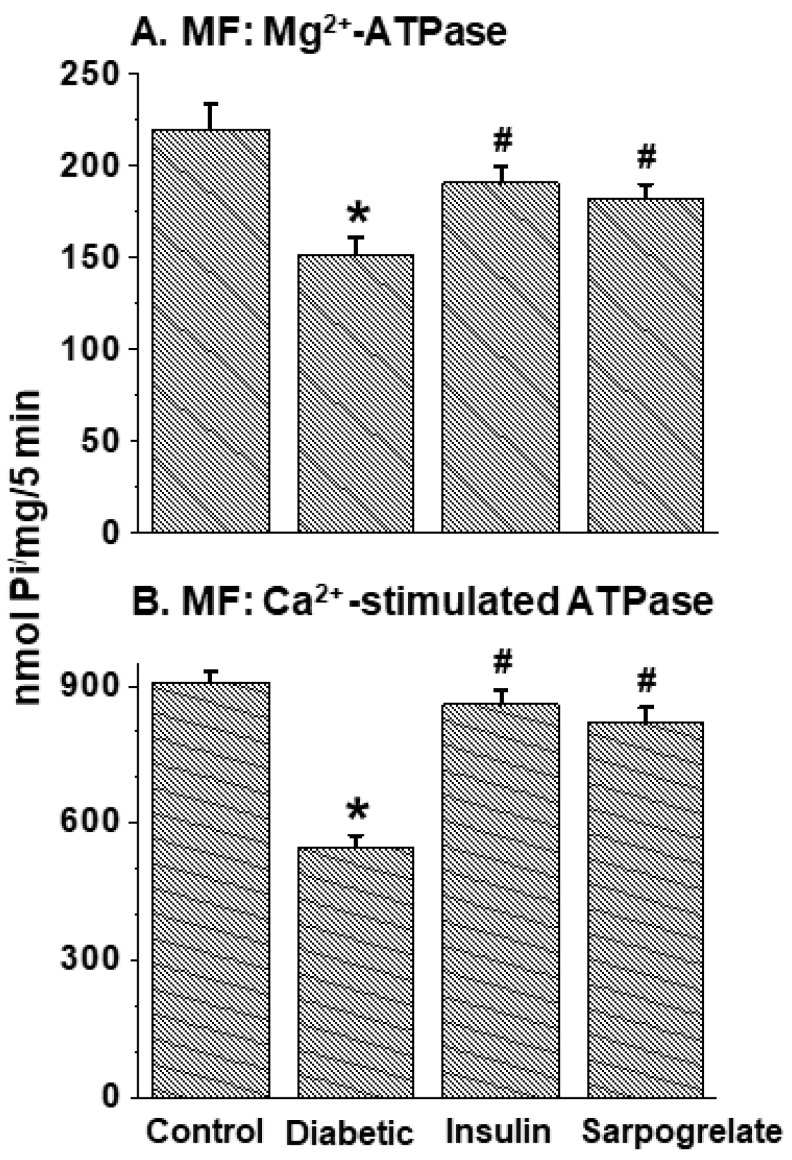
Effects of insulin and sarpogrelate treatments on cardiac myofibrillar (MF) Mg^2+^-ATPase and Ca^2+^-stimulated ATPase activities in diabetic animals. One week after inducing diabetes, rats were treated with insulin (10 units/kg daily) or sarpogrelate (5 mg/kg daily) for 6 weeks. Values are mean ± SE of 4 experiments in each group. *—*p* < 0.05 vs. control; ^#^—*p* < 0.05 vs. diabetic.

**Figure 4:**
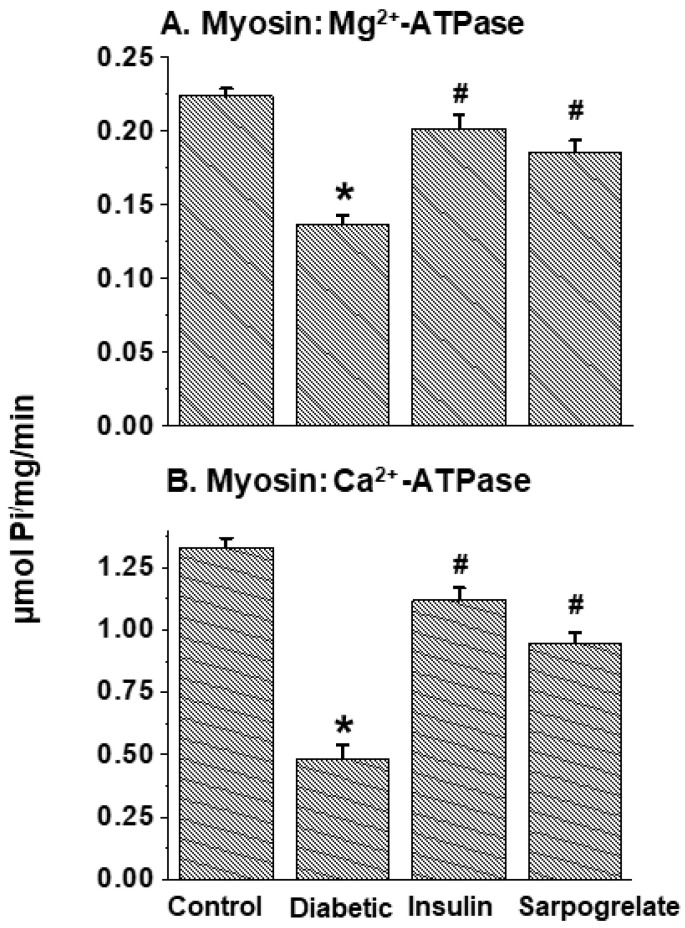
Effects of insulin and sarpogrelate treatments on cardiac myosin Mg^2+^-ATPase and myosin Ca^2+^-ATPase activities in diabetic animals. One week after inducing diabetes, rats were treated with insulin (10 units/kg daily) or sarpogrelate (5 mg/kg daily) for 6 weeks. Values are mean ± SE of 4 experiments in each group. *—*p* < 0.05 vs. control; ^#^—*p* < 0.05 vs. diabetic.

The data in Figure 3 show that both MF Mg^2+^-ATPase and MF Ca^2+^-stimulated ATPase activities were depressed in diabetic hearts; these alterations were fully or partially (but significantly) attenuated by the treatment of diabetic animals with insulin or sarpogrelate. Furthermore, myosin Mg^2+^-ATPase and myosin Ca^2+^-ATPase activities were also decreased in diabetic hearts, but these changes were attenuated only partially (but significantly) by insulin or sarpogrelate treatment (Figure 4). Unlike MF Mg^2+^-ATPase, the beneficial effect of insulin on myosin Mg^2+^-ATPase was somewhat greater (but not significantly) than that of sarpogrelate, whereas that on myosin Ca^2+^-ATPase was significantly (*p* > 0.05) greater than that of sarpogrelate (Figure 3 and Figure 4).

### 3.6. Cardiac Oxidative Stress Biomarkers

In order to gain some information regarding the mechanisms of the beneficial effects of insulin and sarpogrelate treatments on the diabetic heart, some biomarkers of oxidative stress were monitored in control and experimental hearts. The results in Table 6 indicate that malondialdehyde and oxidized glutathione content were increased, whereas reduced glutathione content was decreased in the diabetic heart. Furthermore, the activities of both glutathione peroxidase and superoxide dismutase were depressed in the diabetic heart (Table 6). All of these alterations in oxidative stress biomarkers were attenuated significantly by the treatment of diabetic animals with insulin or sarpogrelate. On the other hand, the activity of catalase in the heart was not affected by diabetes with or without insulin or sarpogrelate treatment (Table 6).

## 4. Discussion

In this study, we have shown that body weight, heart weight and plasma insulin lev-els were decreased, whereas several metabolic parameters, such as plasma glucose, cho-lesterol, free fatty acids and triglyceride levels, were increased in diabetic animals. While heart rate and blood pressure were increased, different parameters of cardiac function, such as LVDP, +dP/dT and −dP/dT, were depressed in diabetic animals without any changes in LVEDP. Such hemodynamic and metabolic alterations are in agreement with our previous observations related to chronic diabetes [32,34].

Furthermore, the treatment of diabetic animals with insulin or sarpogrelate was observed to attenuate diabetes-induced hemodynamic and metabolic changes either fully or partially, indicating that the beneficial effects of sarpogrelate on diabetic animals may involve mechanisms similar to those of insulin. The observed increase in the plasma level of insulin in diabetic animals upon treatment with sarpogrelate is consistent with our finding that this agent prevented the inhibitory effect of the 5-HT-induced release of insulin from the pancreas [32]. Furthermore, the treatment of diabetic animals with sarpogrelate, like insulin, was found to lower plasma glucose levels by increasing the membrane glucose transporter GLUT4 protein content, but this agent, unlike insulin, increased GLUT1 content in the heart [32]. It should be pointed out that sarpogrelate also reduced the elevated levels of glucose in obese mice [45]. Thus, it appears that sarpogrelate produces antidiabetic insulin-like effects to promote glucose utilization, reduce lipid levels and attenuate diabetes-induced defects in heart function both by acting on 5-HT_2A_ receptors and through the release of insulin from the pancreas.

Both creatine phosphate and ATP contents were observed to be decreased, whereas the contents of ADP and AMP were increased in diabetic animals. Such changes in high-energy phosphate stores may be due to the depression of MT state 3 respiration and oxidative phosphorylation activities in the diabetic heart. The observed changes in cardiac energy stores and MT function in chronic diabetes are in agreement with previous reports [34,38]. The depressed activity of oxidative metabolism has also been reported in MT from genetically diabetic mice [46]. On the other hand, the treatment of diabetic rats with sarpogrelate or insulin was found to attenuate diabetes-induced alterations in high-energy phosphate stores as well as changes in MT state 3 respiration and oxidative phosphorylation. It should be pointed out that sarpogrelate has been reported to attenuate ischemia–reperfusion-induced alterations in cardiac high-energy phosphate stores [34]. It is also noted that MT Ca^2+^-uptake and Mg^2+^-ATPase activities in the hearts of animals with chronic diabetes were found to be decreased. Although the treatment of diabetic animals with sarpogrelate or insulin attenuated alterations in MT Ca^2+^-uptake, changes in MT Ca^2+^-ATPase activity were not affected by these treatments. Such depressed changes in MT Mg^2+^-ATPase in chronic diabetes may represent an irreversible state of the MT membrane. In fact, dramatic changes in MT ultrastructure and permeability have been reported to occur in diabetic cardiomyopathy as well as due to ischemia–reperfusion [34,46]. Thus, in view of the well-established role of MT function and energy stores in maintaining cardiac function and structure, it is evident that sarpogrelate, like insulin, may improve cardiac performance in diabetic cardiomyopathy.

Previously, we have reported varying degrees of depression of SL Na^+^-K^+^ ATPase, Na^+^-Ca^2+^ exchange, ATP-dependent Ca^2+^-uptake, Ca^2+^-channel density and Ca^2+^-stimulated ATPase activities, whereas the sensitivity of Ca^2+^ channels was increased and Mg^2+^-ATPase activity was unchanged in the diabetic heart [35,36]. The observed alterations in these SL activities in the hearts of animals with chronic diabetes in this study are similar to these reports. In addition, we have found that the treatment of diabetic animals with sarpogrelate or insulin attenuated diabetes-induced changes in SL activities. In view of the direct or indirect roles of SL Na^+^-K^+^ ATPase, Na^+^-Ca^2+^ exchange, Ca^2+^-uptake, Ca^2+^-stimulated ATPase and Ca^2+^-channel activities in Ca^2+^ entry and Ca^2+^ removal from cardiomyocytes, alterations in these SL activities in the diabetic heart are considered to account for the occurrence of intracellular Ca^2+^ overload, metabolic defects and the development of diabetic cardiomyopathy [18,19]. Thus, the attenuation of diabetes-induced changes in SL activities by both sarpogrelate and insulin treatments may be associated with the beneficial effects of these interventions on myocardial metabolism, cardiac function and cardiac ultrastructure as a consequence of preventing the occurrence of intracellular Ca^2+^ overload. Such antidiabetic actions of sarpogrelate may partly be mediated by the blockade of 5-HT_2A_ receptors [32], which are activated by the elevated levels of 5-HT in diabetes [20,21,22,23].

Chronic diabetes has been reported to depress cardiac SR Ca^2+^-pump and Ca^2+^-release activities, which are considered to explain the impaired relaxation of the heart [18,37]. On the other hand, defects in MF and myosin ATPases have been shown to be associated with depressed cardiac contraction in chronic diabetes [18,39,40,41]. The results of this study regarding depressed SR Ca^2+^-uptake, Ca^2+^-stimulated ATPase and Ca^2+^-release activities without any changes in Mg^2+^-ATPase in the chronic diabetic heart are in agreement with our previous observations [37]. Likewise, the data on changes in cardiac MF Ca^2+^-stimulated ATPase, MF Mg^2+^-ATPase, myosin Ca^2+^-ATPase and myosin Mg^2+^-ATPase in chronic diabetes are also consistent with earlier observations [39]. Furthermore, sarpogrelate and insulin treatments were observed to partially or fully attenuate diabetes-induced alterations in both SR and MF activities. Thus, it is evident that the improvement of cardiac function upon the treatment of diabetic animals with sarpogrelate or insulin may be attributed to a reduction in the SR and MF defects. In view of the marked increase in 5-HT levels in diabetes [20,21,22,23], sarpogrelate might also produce beneficial effects on SR and MF activities by blocking 5-HT_2A_ receptors. It should be pointed out that the activities of both MF and myosin Mg^2+^-ATPase, like that of MT Mg^2+^-ATPase, were depressed in the diabetic heart; however, MF and myosin Mg^2+^-ATPase activities, unlike that of MT Mg^2+^-ATPase, in the diabetic heart were increased significantly by the treatment of diabetic animals with sarpogrelate or insulin. On the other hand, the activities of cardiac SR Mg^2+^-ATPase and SL Mg^2+^-ATPase were unaltered in diabetic animals with or without sarpogrelate or insulin treatment. Such differences in the responsiveness of MF, MT, SL and SR Mg^2+^-ATPases may be due to differences in the structure and function of the enzyme molecules in various subcellular organelles. It should also be mentioned that the beneficial effects of insulin treatment on most of the parameters measured in this study were somewhat greater, but not significantly, than those observed with sarpogrelate treatment. Such differences in the beneficial effects of these treatments may be due to differences in the doses of these interventions.

Earlier, we showed that a well-known 5-HT_2A_ receptor antagonist, sarpogrelate, releases insulin from the pancreas, and its effect in promoting glucose uptake was additive with that insulin [32]. In this study, we observed that this agent produces beneficial effects on hemodynamic, metabolic and subcellular alterations in diabetic animals, which are similar to those seen upon treatment with insulin. In addition to the plasma levels of 5-HT being elevated in diabetes, this hormone has also been reported to induce diabetes-like metabolic alterations [20,21,22,23]. Thus, it is likely that sarpogrelate may exert its effects on diabetes through both the blockade of 5-HT_2A_ receptors and the release of insulin from the pancreas. It should be noted that diabetes is a highly complex disease that is known to affect several systems in the body. Particular effects include the aggregation of platelets for releasing 5-HT, the activation of the sympathetic nervous system for releasing catecholamines and the activation of the renin–angiotensin system for the formation of Ang II in diabetes; all of these vasoactive hormones are known to promote oxidative stress in the diabetic heart [18,19]. In addition, both high levels of plasma glucose and excessive utilization of free fatty acids by MT due to insulin deficiency or insulin ineffectiveness in diabetes have been reported to generate oxidative stress [18,19]. Thus, it appears that the beneficial insulin-like effects of sarpogrelate treatment on hemodynamic, metabolic and subcellular activities in the present study may have occurred as a consequence of a reduction in the development of oxidative stress in diabetic animals. This view is supported by our observations that the treatment of diabetic animals with sarpogrelate or insulin markedly attenuated the altered levels of some biomarkers of oxidative stress, such as malondialdehyde and oxidized glutathione, glutathione peroxidase and superoxide peroxidase, in the diabetic heart. Since the levels of catalase were not changed in the diabetic heart with or without treatment, it is likely that the modification of oxidative stress biomarkers by diabetes, as well as due to insulin or sarpogrelate treatment, is site-specific in the oxidative stress pathway. Nonetheless, on the basis of the attenuation of diabetes-induced defects in SL, SR and MF activities by treatments with vitamin E, propranolol and losartan, it has been indicated that oxidative stress plays a critical role in inducing metabolic and subcellular abnormalities during the development of diabetic cardiomyopathy [18,19]. Accordingly, in view of the results of the present study, as well as our previous observations, it is evident that the improvement of cardiac performance, metabolic defects and alterations in subcellular activities by sarpogrelate or insulin treatment may be a consequence of a reduction in the level of oxidative stress.

## 5. Concluding Remarks

Chronic diabetes is now well known to induce diabetic cardiomyopathy, which is associated with the development of dramatic cardiovascular abnormalities, such as hemodynamic, metabolic, structural and subcellular defects. In this study, we have demonstrated that the treatment of diabetic animals with sarpogrelate, a 5-HT_2A_ receptor blocker, produced beneficial effects on diabetes-induced alterations in cardiac performance, plasma glucose and lipid levels, myocardial high-energy phosphate stores and the functional activities of subcellular organelles in the heart. These antidiabetic actions of sarpogrelate were similar to those observed with the treatment of diabetic animals with insulin. While the beneficial effect of sarpogrelate treatment on MT oxidative phosphorylation activity seems to be associated with preventing the depression of both creatine phosphate and ATP stores, the antidiabetic action of this intervention on SR and SL Ca^2+^-handling activities, as well as on MF Ca^2+^-stimulated ATPase activity, may explain the improvement of cardiac function in the diabetic heart. Furthermore, the depression of plasma glucose and lipid levels in diabetic animals by both sarpogrelate and insulin treatments may represent a shift in substrate utilization by MT to maintain the energy status of the heart as well as to reduce the development of oxidative stress in diabetes. Thus, in view of such observations in this study, it is suggested that sarpogrelate may prove to be a novel therapy for preventing diabetes-induced complications such as diabetic cardiomyopathy. However, extensive biomedical research and clinical work with this intervention need to be carried out before making any definitive conclusions.

## Figures and Tables

**Table 1:** Effects of insulin and sarpogrelate treatments on general characteristics of diabetic animals.

Parameters	Control	Diabetic	Diabetic+	Diabetic+
			Insulin-Treated	Sarpogrelate-Treated
Body wt (g)	520 ± 18.9	325 ± 16.7 *	440 ± 20.4 ^#^	412 ± 18.6 ^#^
Heart wt (mg)	1404 ± 26.7	1138 ± 18.5 *	1305 ± 22.7 ^#^	1230 ± 15.8 ^#^
Plasma glucose (mM)	7.6± 0.8	33.6± 2.4 *	6.7± 2.5 ^#^	26.3 ± 1.7 ^#^
Plasma insulin (ng/mL)	0.58± 0.03	0.24 ± 0.04 *	0.61 ± 0.04 ^#^	0.47 ± 0.05 ^#^
Plasma cholesterol (nM)	1.54 ± 0.03	2.58 ± 0.03 *	1.72± 0.04 ^#^	1.90 ± 0.09 ^#^
Plasma FFAs (m-eq/L)	0.29 ± 0.02	0.44 ± 0.04 *	0.26 ± 0.03 ^#^	0.31 ± 0.02 ^#^
Plasma TGs (mM)	2.58 ± 0.36	6.75 ± 0.49 *	2.57 ± 0.48 ^#^	3.89 ± 0.59 ^#^

Streptozotocin (65 mg/kg)-induced diabetic rats after one week were treated with insulin (10 units/kg daily) or sarpogrelate (5 mg/kg daily) for 6 weeks. Values are mean ± S.E. of 6 to 8 animals in each group. * *p* < 0.05 compared to control; ^#^ *p* < 0.05 compared to diabetic. Abbreviations: FFAs, free fatty acids; TGs, triglycerides.

**Table 2:** Effects of insulin and sarpogrelate treatments on hemodynamic parameters in diabetic animals.

Parameters	Control	Diabetic	Diabetic+	Diabetic+
			Insulin-Treated	Sarpogrelate-Treated
Heart rate (beats/min)	412 ± 21.4	316± 14.3 *	392 ± 16.6 ^#^	372 ± 12.8 ^#^
Blood Pressure (mm/Hg)	111 ± 8.9	140 ± 6.8 *	106 ± 7.7 ^#^	102 ± 6.9 ^#^
LVDP (mmHg)	110.2 ± 4.0	86.9 ± 2.1 *	99.9 ± 5.7 ^#^	111.3 ± 3.6 ^#^
LVEDP (mmHg)	3.6 ± 0.2	3.7 ± 0.3	3.7 ± 0.2	3.8 ± 0.3
+dP/dt (mmHg/s)	9421 ± 975	7915 ± 723 *	9506 ± 794 ^#^	* 9744 ± 242 ^#^
−dP/dt (mmHg/s)	8874 ± 627	6685 ± 93 *	9011 ± 662 ^#^	9496 ± 468 ^#^

Streptozotocin (65 mg/kg)-induced diabetes in rats for 1 week, followed by 6 weeks of treatment with insulin (10 units/kg daily) or sarpogrelate (5 mg/kg daily). LVDP, left ventricular developed pressure; LVEDP, left ventricular end-diastolic pressure; +dP/dt, rate of contraction; −dP/dt, rate of relaxation. Values are mean ± S.E. of 6 to 8 animals in each group. * *p* < 0.05 compared to control; ^#^ *p* < 0.05 compared to diabetic.

**Table 3:** Effects of insulin and sarpogrelate treatments on cardiac high-energy stores in diabetic animals.

Parameters	Control	Diabetic	Diabetic+	Diabetic+
			Insulin-Treated	Sarpogrelate-Treated
CP (µmol/g)	6.58 ± 0.30	3.66 ± 0.48 *	5.79 ± 0.28 ^#^	5.24 ± 0.36 ^#^
ATP (µmol/g)	4.82 ± 0.21	3.58 ± 0.15 *	4.46 ± 0.19 ^#^	4.16 ± 0.14 ^#^
ADP (µmol/g)	1.36 ± 0.07	1.91 ± 0.08 *	1.46 ± 0.05 ^#^	1.58 ± 0.09 ^#^
AMP (µmol/g)	0.51 ± 0.03	0.97 ± 0.06 *	0.65 ± 0.04 ^#^	0.72 ± 0.05 ^#^

One week after inducing diabetes, rats were treated with insulin (10 units/kg daily) or sarpogrelate (5 mg/kg daily) for 6 weeks. Values for high-energy phosphate stores are mean ± SE of 4 animals in each group. Abbreviation: CP, creatine phosphate. *—*p* < 0.05 vs. control; ^#^—*p* < 0.05 vs. diabetic.

**Table 4:** Effects of insulin and sarpogrelate treatments on cardiac mitochondrial (MT) oxidative phosphorylation, Ca^2+^-uptake and ATPase activities.

Parameters	Control	Diabetic	Diabetic+	Diabetic+
			Insulin-Treated	Sarpogrelate-Treated
A. MT oxidative phosphorylation:				
ADP/O ratio	2.86 ± 0.23	2.59 ± 0.31	2.74 ± 0.24	2.86 ± 0.25
State 3				
(natoms O/mg/min)	192 ± 8.3	144 ± 9.6 *	182 ± 6.9 ^#^	174 ± 7.7 ^#^
State 4				
(natoms O/mg/min)	17.5 ± 1.8	19.4 ± 2.7	20.3 ± 2.5	18.2 ± 2.5
Oxidative phosphorylation rate				
(State 3x ADP/O ratio)	549 ± 34	373 ± 42 *	498 ± 31 ^#^	497 ± 37 ^#^
B. MT Ca^2+^ uptake and ATPase:				
Ca^2+^ uptake	134 ± 6.2	84 ± 3.6 *	121 ± 4.9 ^#^	106 ± 3.8 ^#^
Mg^2+^ ATPase	9.6 ± 0.4	6.2 ± 0.4 *	6.4 ± 0.3	6.0 ± 0.3
(µmol Pi/mg/5 min)				

One week after inducing diabetes, rats were treated with insulin (10 units/kg daily) or sarpogrelate (5 mg/kg daily) for 6 weeks. The ADP/O ratio was calculated as nmol ADP phosphorylated per natoms O_2_ consumed. Oxidative phosphorylation was measured by using 5 mM glutamate as a substrate. Ca^2+^-uptake activity was determined in the presence of 10 µM ^45^Ca and is expressed in nmol Ca^2+^/mg/5 min. Mg^2+^-ATPase activity is expressed in umol Pi/mg/5 min. Values are mean ± SE of 6 animals in each group. *—*p* < 0.05 vs. control; ^#^—*p* < 0.05 vs. diabetic.

**Table 5:** Effects of insulin and sarpogrelate treatments on cardiac sarcolemma (SL) Ca^2+^-channel, Ca^2+^-uptake and ATPase activities as well as sarcoplasmic reticulum (SR) Ca^2+^-stimulated ATPase and Mg^2+^-ATPase activities in diabetic animals.

Parameters	Control	Diabetic	Diabetic+	Diabetic+
			Insulin-Treated	Sarpogrelate-Treated
A. SL Ca^2+^ channel and Ca^2+^ transport:				
Ca^2+^-channel density	154 ± 13	95 ± 10 *	149 ± 12 ^#^	131 ± 9 ^#^
(fmol/mg)				
Kd (nM)	0.34 ± 0.03	0.24 ± 0.02 *	0.41 ± 0.09 ^#^	0.32 ± 0.02 ^#^
ATP-dependent Ca^2+^ uptake				
(nmol/mg/min)	15.6 ± 1.6	6.4 ± 0.9 *	11.7 ± 1.2 ^#^	10.1 ± 0.7 ^#^
Ca^2+^-stimulated ATPase				
(µmol Pi/mg/5 min)	1.6 ± 0.3	0.7 ± 0.1 *	1.5 ± 0.2 ^#^	1.2 ± 0.2 ^#^
Mg^2+^-ATPase				
(µmol Pi/mg/5 min)	14.2 ± 2.1	13.7 ± 2.6	15.1 ± 2.8	14.7 ± 3.3
B. SR Ca^2+^-stimulated ATPase and Mg^2+^-ATPase:				
Ca^2+^-stimulated ATPase				
(µmol Pi/mg/5min)	3.7 ± 0.49	2.5 ± 0.24 *	3.4 ± 0.24 ^#^	3.2 ± 0.16 ^#^
Mg^2+^-ATPase				
(µmol Pi/mg/5 min)	10.6 ± 0.4	9.6 ± 0.5	10.3 ± 0.4	9.8 ± 0.3

One week after inducing diabetes, rats were treated with insulin (10 units/kg daily) or sarpogrelate (5 mg/kg daily) for 6 weeks. Values are mean ± SE of 4 experiments in each group. *—*p* < 0.05 vs. control; ^#^—*p* < 0.05 vs. diabetic.

**Table 6:** Effects of insulin and sarpogrelate treatments on some cardiac biomarkers of oxidative stress in diabetic animals.

Parameters	Control	Diabetic	Diabetic+	Diabetic+
			Insulin-Treated	Sarpogrelate-Treated
Malondialdehyde				
(nmol/g heart)	80 ± 8.5	106 ± 6.2 *	83 ± 5.1 ^#^	90 ± 4.4 ^#^
Reduced glutathione				
(ng/mg protein)	258 ± 10.2	164 ± 9.5 *	221 ± 7.9 ^#^	203 ± 6.8 ^#^
Oxidized glutathione				
(ng/mg protein)	72 ± 5.4	125 ± 7.2 *	86 ± 5.6 *	92 ± 4.3 ^#^
Glutathione peroxidase				
(nmol/mg protein/min)	82 ± 5.8	60 ± 4.2 *	87 ± 6.1 ^#^	76 ± 3.4 ^#^
Superoxide dismutase				
(Units/mg protein)	10.4 ± 1.2	4.8 ± 1.3 *	8.6 ± 1.7 ^#^	6.9 ± 0.8 ^#^
Catalase				
(Units/mg protein)	23.6 ± 1.7	26.4 ± 1.9	28.1 ± 2.3	24.4 ± 1.8

One week after inducing diabetes, rats were treated with insulin (10 units/kg daily) or sarpogrelate (5 mg/kg daily) for 6 weeks. Values are mean ± SE of 4 animals in each group. *—*p* < 0.05 vs. control; ^#^—*p* < 0.05 vs. diabetic.

## Data Availability

Data are contained within the article.
